# Complexity of Antibiotic Resistance in Commensal *Escherichia coli* Derived from Pigs from an Intensive-Production Farm

**DOI:** 10.1264/jsme2.ME17041

**Published:** 2018-09-29

**Authors:** Justyna Mazurek, Ewa Bok, Katarzyna Baldy-Chudzik

**Affiliations:** 1 Department of Microbiology and Genetics, Faculty of Biological Sciences, University of Zielona Góra Monte Cassino 21b, 65–561 Zielona Góra Poland

**Keywords:** antibiotic resistance, commensal *Escherichia coli*, food production animals

## Abstract

Antibiotics in animal husbandry are used to maintain welfare, but lead to the generation of resistant strains. We analyzed commensal multidrug-resistant *Escherichia coli* from pigs at the beginning and end of the production cycle in a farm with a farrow-to-finish system in order to investigate whether clonal spread or horizontal gene transfer constitutes the main factor responsible for the prevalence of resistance in this environment. Among 380 isolates, 56 multidrug-resistant *E. coli* with a similar resistant phenotype were selected for more detailed investigations including a genomic similarity analysis and the detection of mobile elements. Isolates carried *bla_TEM-1_*, *aadA1*, *strA/B*, *tetA*, *tetB*, *tetC*, *dfrA1*, *dfrA5*, *dfrA7*, *dfrA12*, *sul1*, *sul2*, *sul3*, and *qnrS* resistance genes, with the common co-occurrence of genes encoding the same resistance phenotype. A pulse-field gel electrophoresis analysis of the genomic similarity of multidrug-resistant *E. coli* showed ≤65% similarity of most of the tested strains and did not reveal a dominant clone responsible for the prevalence of resistance. Class 1 and 2 integrons and transposons 7 and 21 were detected among mobile elements; however, some were truncated. Plasmids were represented by 11 different incompatibility groups (K, FIB, I1, FIIA, FIC, FIA, Y, P, HI1, B/O, and T). Genetic resistance traits were unevenly spread in the clonal groups and suggested the major rearrangement of genetic material by horizontal gene transfer. The present results revealed that in commensal *E. coli* from pigs in a homogeneous farm environment, there was no dominant clone responsible for the spread of resistance and persistence in the population.

The potential contribution of food-producing animals to the development of antibiotic resistance in bacteria has been widely discussed (16, 17, 18, 38). Animal breeding farms constitute specific environments with large concentrations of animals at various stages of the production cycle, necessitating the use of antibiotics, particularly for young pigs in the post-weaning period (8, 15). Although antibiotic administration is directed against pathogenic bacteria, commensals are also affected. Thus, the commensal intestinal flora reflects the history of antibiotic usage and constitutes a specific reservoir of resistant strains. An epidemiological threat may arise, particularly from multidrug-resistant strains that accumulate resistance genes (40). The European Food Safety Authority indicates commensal species, such as *Escherichia coli*, as indicator organisms for monitoring resistance to antibiotics in animals (12).

We herein investigated the resistance of *E. coli* derived from pigs from one intensive-production farm, working in a farrow-to-finish system. The aim of the present study was to elucidate the mechanisms responsible for the prevalence of *E. coli* resistance in animals from this environment. The research hypothesis assumed the occurrence of two possible mechanisms of resistance dissemination. The first assumption was that if the administration of antibiotics to piglets generates specific *E. coli*-resistant clones, they will also be present in 5-month-old adult sows at the end of the production cycle. The second mechanism concerned the process of horizontal gene transfer. If horizontal gene transfer is mostly responsible for the prevalence of resistance, mobile genetic elements will be spread among *E. coli* strains in young as well as adult animals.

Therefore, *E. coli* was collected from piglets at the beginning of the production cycle and from sows at the end of the production cycle. This sampling plan provided the opportunity to assess whether a particular multidrug-resistant clone persists in a given environment or if resistance determinants are spread by horizontal gene transfer between the different strains. The clonal diversity of strains was assessed by a PFGE analysis, and the contribution of horizontal gene transfer to resistance development was investigated by the identification of mobile elements, such as plasmids and the most prevalent transposons (7 and 21), class 1 and 2 integrons, and insertion sequences in the environment (1, 10, 21, 37).

## Materials and Methods

### Characteristics of the farm and animals

The selected farm is located in the western part of Poland. It is an intensive-production farm with a farrow-to-finish production system. Approximately 55,000 piglets are born annually. Groups of animals are housed in separate buildings for the different phases of their life and moved to another building. Animals enter a freshly disinfected environment, complying with all-in/all-out (AIAO) systems. Buildings are in close proximity to one another in order to ensure a uniform microclimate and the health status of all groups of pigs. Each group of pigs is fed a different diet.

### Sample collection, isolation, and identification of *E. coli*

Groups of animals that had passed the same metaphylactic program were selected, and represented the main animal groups from the farm. There were four litters of post-weaning piglets aged 5–10 weeks (220 animals) that were in the successive stages of the metaphylactic program (in the five consecutive weeks, sulfonamide, trimethoprim, amoxicillin, and sulfaguanidine were administered in medicated feed) and two herds of sows (160 animals). Sows underwent the same metaphylactic program and were from between 10 and 18 weeks after antibiotic treatment. The program was introduced for piglets after weaning based on the diagnosis of streptococcosis and colibacillosis. Fresh fecal samples were collected only once and *E. coli* was selected as described previously (29). One isolate per pig fecal sample was randomly chosen for further study.

### Antibiotic sensitivity testing

Antibiotic sensitivity to 11 antimicrobial agents—ampicillin, cefotaxime, ceftazidime, tetracycline, streptomycin, gentamicin, nalidixic acid, ciprofloxacin, sulfamethoxazole, trimethoprim, and chloramphenicol—was assessed using the broth microdilution method with Sensititre plates for veterinary application (TREK Diagnostic Systems, Independence, OH, USA). The results obtained were interpreted according to the epidemiological MIC cut-off values set by EFSA (13). *E. coli* ATCC 25922 was used as a susceptibility control strain.

### Antibiotic resistance gene identification

All isolates were examined for the presence of genes encoding resistance to β-lactams: *bla_TEM_*, *bla*_SHV_ (24), *bla*_CTX-M_ (22), and *ampC* (31); aminoglycosides: *aadA1*, *strA/B*, and *aac(*3*)-IV* (24), tetracycline: *tetA*, *tetB*, *tetC*, *tetD*, and *tetM* (30) sulfamethoxazole: *sul1*, *sul2*, and *sul3* (24) trimethoprim: *dfrA1*, *dfrA5*, *dfrA7*, and *dfrA12* (14); chloramphenicol: *catA1*, *cmlA1*, and *floR* (41) and quinolones: *qnrA1-6*, *qnrB1-6*, and *qnrS1-2* (6) by multiplex and simplex PCR amplification.

### Pulse-field gel electrophoresis (PFGE)

A PFGE analysis was performed with a CHEF-MAPER system (Bio-Rad, Hercules, CA, USA), according to the PulseNet protocol (http://www.cdc.gov/pulsenet/pathogens/pfge.html) with XbaI digestion (7). Based on PFGE fingerprinting patterns, a dendrogram was created using the UPGMA method with the Dice similarity coefficient (Fingerprinting II, V 3.0; Bio-Rad). The genetic relatedness of tested *E. coli* was assessed and isolates showing more than 90% PFGE pattern similarity were considered to belong to the same clonal group (36).

### Mobile genetic element identification

The presence of class 1 and class 2 integrons was analyzed by the PCR identification of their elements: integrase genes (*int1* and *int2*), variable regions containing gene cassettes (c1gc and c2gc) and the 3′ conserved region of the class 1 integron (*qacEΔ1* and *sul1*) (22). Gene cassettes were identified by the sequencing of PCR products (Genomed, Poland). Transposons 7 (Tn7) and 21 (Tn21) were identified by the amplification of genes encoding their individual components. Transposase (*tnp*A) and resolvase (*tnpR*) genes, the regulatory gene *merR*, and one of the structural Genes, *merA*, were identified in Tn21. In Tn7, all transposase genes—*tnsA*, *B*, *C*, *D*, and *E*—were identified using multiplex PCR (Supplementary Table S1). Furthermore, the mobile elements’ common regions—ISCR1, ISCR2, and ISCR3—were detected (Table S1). Plasmid replicon typing was conducted according to the PCR method designed by Carattoli (4).

### Statistical analysis

Pearson’s chi-squared test was used to assess the relationships between the prevalence of resistance genes and the source of the *E. coli* origin with the significance level set at *P*<0.05 (statistical program R version 2.15.0).

## Results

Antibiotic susceptibility tests on 380 *E. coli* isolates revealed a high prevalence of resistance among isolates from pigs. In isolates from piglets at the beginning of the production cycle during the administration of antibiotics, the frequency of resistant *E. coli* was 99%. Among isolates from adult pigs at the end of the production cycle, resistance was 100%. *E. coli* was resistant not only to the antibiotics used in the metaphylactic program, but also to other agents. The dominant patterns of phenotypic resistance were as follows: AMP/STR/TET/TMP/SMX, STR/TET/TMP/SMX, and AMP/STR/GEN/TET/TMP/SMX/CIP. Fourteen different resistance genes (*bla_TEM-1_*, *aadA1*, *strA/B*, *tetA*, *tetB*, *tetC*, *dfrA1*, *dfrA5*, *dfrA7*, *dfrA12*, *sul1*, *sul2*, *sul3*, and *qnrS*) were found in the resistant isolates. Among the *E. coli* tested, multidrug isolates with a similar phenotype of resistance and similar set of resistance genes were selected for detailed genetic studies. This set included 56 isolates—34 from piglets (marked as P) and 22 from sows (marked as S). Among these isolates, resistance to β-lactam antibiotics was identified in 41 *E. coli*, which constituted 73% of the tested set (*i.e.*, 41/73%). Only the *bla_TEM_*_-1_ resistance gene was detected in these ampicillin-resistant isolates (Table 1). No gene was detected in four isolates from sows, which were resistant to ampicillin, and one isolate from piglets had the *bla_TEM_*_-1_ gene, but was sensitive to ampicillin (Table 1).

The *aadA1* (45/80%) and *strA/B* (18/32%) genes were present in streptomycin-resistant isolates (50/89%), and their co-occurrence was observed in 13 (23%) *E. coli*. In tetracycline-resistant isolates (40/71%), the *tetA* gene was the most common determinant (30/54%) found in isolates resistant to tetracycline, followed by the *tetC* and *tetB* genes (in 8/14% of isolates each). The co-occurrence of two *tet* genes was observed in three *E. coli*, while no gene was detected in three resistant isolates. Four *dfrA* genes were detected among trimethoprim-resistant isolates (56 strains/100%), and the *dfrA1* gene was the most prevalent (present in 49 isolates/87%), followed by *dfrA12* (35/62.5%), *dfrA5* (19/34%), and *dfrA7* (18/32%). The presence of two to three *dfrA* genes in various combinations was detected in 98% of the isolates tested. Three different *sul* genes were detected among 54 sulfamethoxazole-resistant isolates. The *sul1* gene was the most prevalent (present in 36 isolates/64%), and the *sul2* and *sul3* genes occurred at similar frequencies (25/45%; 24/43%, respectively). The occurrence of more than one *sul* gene was found in 27 isolates (48%), mainly in strains from piglets. Regarding quinolone resistance, the *qnrS* gene was identified in three isolates from piglets.

**Fig. 1:**
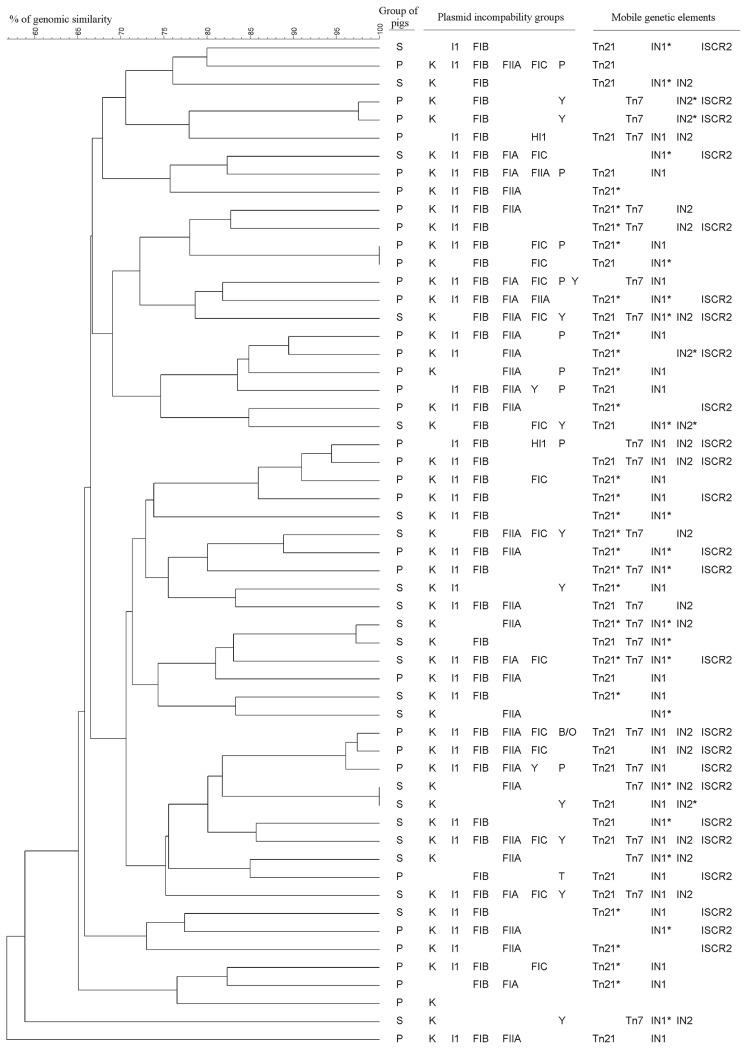
Dendrogram of the relationship between PFGE patterns of 56 multidrug resistant *E. coli* isolates.

In order to assess whether any dominant *E. coli* clones carrying resistance determinants existed in the tested population, the genetic diversity of multidrug-resistant strains was examined using a PFGE analysis. The UPGMA dendrogram created based on the PFGE genomic patterns of 56 resistant *E. coli* showed the presence of two major heterogeneous groups with a similarity of 65% (Fig. 1). The first group comprised 22 isolates derived mainly from piglets (17 *E. coli*). The second group was formed by 26 isolates—15 from adult pigs and 11 from piglets. Eight isolates (6 from piglets and 2 from adults) were not classified in the main groups. In six cases, two to three individual isolates were characterized by identical genetic profiles (>90% similarity) and were derived from the same group of pigs. In the next step, the spread of resistance genes in particular similarity groups of the dendrogram was analyzed. Resistance genes were evenly distributed in these groups.

In order to identify mobile genetic platforms with the ability to carry resistance genes, the presence of integrons, transposons, and common regions was investigated. In class 1 integron typing, both of the typical integrons with a complete set of elements and atypical (incomplete) integrons without one of the elements were detected in 44 *E. coli* (Table 2). Typical integrons were the most prevalent in *E. coli* from piglets (*P*=0.0027), while atypical class 1 integrons were identified significantly more often among *E. coli* isolated from sows (*P*=0.0389). In class 1 integrons, four gene cassette arrays were detected: *dfrA1-aadA1* (in 20 isolates/36%), *aadA1* (3/5%), *dfrA7* (2/4%), and *dfrA12-aadA2* (1/2%). Class 2 integrons were detected in 22/39% of all tested isolates and contained two gene cassette arrays: *dfrA1-sat2-aadA1* (11/20%) and *estX-sat2-aadA1* (5/9%) (Table 2). Atypical structures were also found in this class of integrons (Table 2). Class 1 integrons were more frequently detected than class 2 integrons among *E. coli* from each group of animals. The co-existence of class 1 and 2 integrons was observed in 14 *E. coli* isolates.

In the case of transposons, their individual components, commonly accepted marker genes, were identified. Transposases (*tnp*) and mercury resistance operon genes (*mer*) of transposon 21 were both detected in 22 isolates (39%) (Table 3). Only *tnp* genes were detected in 7 isolates (12.5%), while only *mer* genes were detected in the next 16 isolates (29%). Fragments of Tn21 were identified more frequently in strains from piglets than from sows (*P*=0.0218). The presence of Tn7, with all marker genes (*tns A*, *B*, *C*, *D*, and *E*), was detected in 22 (39%) of the tested isolates. The common region ISCR2 was found in 24 isolates (43%) (Table 3), and more frequently in *E. coli* from piglets (*P*=0.0143). Taking into account the dissemination of mobile elements in the similarity groups of the dendrogram, they were spread among all similarity PFGE groups and were not clustered in any of the selected groups (Fig. 1).

The presence of the plasmids was examined by the detection of their replicon types, determining their affiliation to incompatibility groups (Inc). In plasmid replicon typing, 11 different incompatibility groups (K, FIB, I1, FIIA, FIC, FIA, Y, P, HI1, B/O, and T) were detected (Table 4). Replicons belonging to the incompatibility groups K and IncF (FIB, FIIA, I1) were the most frequently identified. Replicons of the incompatibility groups P, HI1, B/O, and T were only found in isolates from piglets. Furthermore, replicons with the FIB, I1, and FIIA groups occurred more frequently among strains from these animals (*P*=0.0113, *P*=0.0035, and *P*=0.0499, respectively).

The replicon co-existence of more than one incompatibility group was found in many isolates. In the analyzed set of 56 strains, 30 different replicon arrangements were detected, representing 2 to 7 incompatibility groups. Three combinations of replicon types K/I1/FIB/FIIA, K/I1/FIB, and K/FIB/FIC occurred in isolates from both groups of animals (Fig. 1). One to three replicons were identified significantly more often in isolates from sows (*P*=0.0343), while 4 or more co-existing replicons were detected more often in *E. coli* from piglets (*P*=0.0218). A correlation was not observed between replicon types and clonal groups in the dendrogram (Fig. 1). Plasmids of the same Inc groups were not related to specific resistance genes or stable platform integrin transposons. Isolates with identical PFGE restriction patterns (6 pairs) had very similar arrangements of resistance traits in one case only.

## Discussion

The present study focused on elucidating whether clonal spreading or horizontal gene transfer is responsible for the prevalence and maintenance of resistance in the closed system of animal husbandry. In the population of animals studied, multidrug-resistant strains were isolated from young post-weaning piglets that received antibiotics and from adult pigs finishing the production cycle. Most of the resistant strains from both groups carried similar resistance genes and mobile elements. However, the PFGE analysis did not show identical genomic patterns, and they were not clustered in a similarity group. These results excluded the hypothesis of the spread of resistant *E. coli* clones in the bacterial population.

The diversity of resistance genes associated with the resistance phenotypes was high, indicating the dynamics of resistance development. The accumulation of resistant genes responsible for a particular antibiotic was significant. The co-existence of genes affecting resistance to the same antibiotic in a single strain was the most prevalent in *E. coli* from piglets that were under antibiotic pressure. This may have resulted from a mutation leading to gene diversity and the accumulation of resistance genes forced by horizontal gene transfer. The resistance genes identified in the *E. coli* tested have also been detected by other researchers. However, the prevalence of strains carrying multiple genes for resistance to one antibiotic was higher in the present study, particularly for trimethoprim-resistance genes, than previously reported (3, 11, 14, 16, 19, 23, 33, 35, 39). It is important to note that the *bla_CTX-M_*, *bla_SHV_*, and *bla_CMY-2_* genes, encoding extended spectrum β-lactamases (ESBLs) (32), were not detected among the strains tested. These genes, which have an origin in a clinical environment, may not have been introduced into the gene pool of the tested environment, although the gene reservoir in farm animals occurs in Poland (26).

The genes responsible for resistance to ampicillin and tetracycline were not found in several strains, suggesting that other resistance determinants are present among the tested strains. It emphasizes the high diversity of the genetic background of resistance in a given environment. Moreover, the presence of resistance genes in sensitive strains indicates high mutation variability. In similar studies, we reported that the trimethoprim resistance genes *dfr* were not expressed due to defective promoters (29). Furthermore, similar changes may have occurred in this case. The occurrence of horizontal gene transfer also strongly indicates rearrangements within mobile elements, such as integrons and transposons. Numerous genetic elements responsible for the transfer of resistance genes were detected among the strains tested. Class 1 and/or class 2 integrons carried gene cassettes that are globally disseminated and have been reported in the literature for years (9, 16, 19, 21, 28). However, in the tested population, integrons were present not only in the form of a typical genetic platform, containing characteristic integron components, but also as atypical integrons (without a variable region of gene cassettes and/or gene of 3′ ends). The gene cassettes in integrons are generally incorporated or excised (“cut-and-paste” relocalization) by site-specific recombination catalyzed by the integrase (34). In the present study, the difference observed in the prevalence of typical and incomplete integrons suggests the strong activity of rearrangement mechanisms, involving the insertion of integron gene cassettes in the course of antibiotic pressure and/or cutting out of gene cassettes when antibiotic pressure ceases. Gene cassettes are genetic elements incapable of self-replication and require the presence of the integron Pc promoter for their expression (10). The consequence of this system is that the transfer of the cassette beyond the promoter may result in the inactivity of the genes.

The presence of atypical class 1 and 2 integrons has been reported, but in a smaller number of isolates (1, 16, 23, 27, 28). The present study is one of the few that allowed for indications of changes in the structures of integrons in strains from a defined population when the pressure of antibiotics occurs and ceases.

Integrons may be transmitted between bacterial cells when they are within larger genetic platforms—in transposons. The presence of Tn7 and Tn21 was detected in the tested set of multidrug-resistant strains. All characteristic genes were detected for Tn7, whereas the complete set of genes and just the 3′ or 5′ ends of genes were detected for Tn21. Fragments of Tn21 were detected more frequently in strains from animals during antibiotic therapy. This is further evidence for the significant rearrangements of mobile genetic elements under antibiotic pressure. The present study also revealed the presence of the common region ISCR2, which was mainly identified among *E. coli* animals during the antibiotic treatment. This group of insertion sequences has been identified previously in association with a number of antibiotic resistance genes, such as *sul2*, *dfr18*, and *floR* (37); however, a correlation was not observed with any of the resistance genes detected in the tested *E. coli* set.

Furthermore, the plasmids appeared to be an important factor responsible for the occurrence of resistance in the studied population. Plasmids from the IncF group were the most commonly identified, which confirms the common observation that plasmids from the IncF family play a significant role in the dissemination of antibiotic resistance in *Enterobacteriaceae* (5, 20). However, higher diversity was noted in the numbers and types of replicon incompatibility groups, particularly among *E. coli* from piglets. This result indicates the important role of plasmids in the transmission of resistance traits in the bacterial population when antibiotics are administered. Plasmids were also present in all multidrug-resistant strains from pigs at the end of the production cycle. Plasmid maintenance in the bacterial population, regardless of environmental pressure, may be the result of plasmid stability mechanisms in cells (2).

The presence of specific (definite) plasmid replicons in strains belonging to the different PFGE similarity groups and the lack of a connection to particular clonal groups also support the hypothesis of the non-clonal dissemination of a resistant determinant among the tested *E. coli*. Strains with a similar Inc plasmid carried a different set of resistance genes, and it was not possible to connect a given gene to a particular plasmid. This indicates the great potential for the dissemination of resistant traits in the farm environment tested.

The present results suggest the existence of a diverse gene pool of resistance; however, it is limited by the closed farm environment. Antibiotic pressure triggers the mechanisms of horizontal gene transfer, which leads to the major rearrangement of genetic material. Classical patterns of mobile elements associated with resistance are as follows: gene cassettes— integrons—transposons—plasmids. These typical schemes are often revealed in clinical strains, with epidemiological significance (25). These elements were found to be reshuffled in the environment tested. There was no single specific genetic platform or one selected environment-specific *E. coli* clone that may be responsible for the resistance phenotype in the analyzed strains. In conclusion, the present results demonstrated that animal farms, in which antibiotics are constantly administered at the early stage of the pig production cycle, constitute a fundamental environment for dynamic changes and the development of antibiotic resistance. This environment is the crucible in which various factors of resistance are mixed, and the driving force is the use of antibiotics. The gene pool changes, but is retained and circulates.

## Supplementary Material



## Figures and Tables

**Table 1:** Prevalence of *E. coli* resistance and resistance genes in multidrug-resistant *E. coli* isolated from groups of pigs.

Antibiotic Agent	Resistance gene	No. (%) of *E. coli* isolated from		

		Piglets (*n*=34)	Sows (*n*=22)	Total (*n*=56)
Ampicillin	resistance	31 (91)	10 (45)	41 (73)

	*bla_TEM-1_*	32 (94)	6 (27)	38 (68)

Streptomycin	resistance	32 (94)	18 (82)	50 (89)

	*aadA1*	26 (76)	19 (50)	45 (80)
	*strA/B*	12 (35)	6 (27)	18 (32)

Tetracycline	resistance	28 (82)	12 (54)	40 (71)

	*tetA*	22 (65)	8 (36)	30 (54)
	*tetB*	1 (3)	7 (32)	8 (14)
	*tetC*	7 (20)	1 (4)	8 (14)

Trimethoprim	resistance	34 (100)	22 (100)	56 (100)

	*dfrA1*	29 (85)	20 (91)	49 (87)
	*dfrA5*	17 (50)	2 (9)	19 (34)
	*dfrA7*	11 (32)	7 (32)	18 (32)
	*dfrA12*	16 (47)	19 (86)	35 (62.5)

Sulfamethoxazole	resistance	34 (100)	20 (91)	54 (96)

	*sul1*	25 (73)	11 (50)	36 (64)
	*sul2*	18 (53)	7 (32)	25 (45)
	*sul3*	17 (50)	7 (32)	24 (43)

Ciprofloxacin	resistance	4 (12)	0	4 (7)

	*qnrS*	3 (9)	0	3 (5)

The presence of the following genes was not detected in the tested set: resistance to β-lactams: *bla_SHV-1_*, *bla_CTX-M_* and *ampC,* tetracycline *tetD;* gentamicin *aac(3)IV*, trimethoprim *dfrA17*, phenicols: *floR*, *cmlA, cat1*; quinolones *qnrA.*

**Table 2:** Prevalence of class 1 and 2 integrons among multidrug-resistant *E. coli* isolated from groups of pigs.

Source of isolates	No. (%) *E. coli* with **class 1 integrons**	Class 1 integron elements			No. (%) *E. coli* with **class 2 integrons**	Class 2 integron elements	
		5′CS*	variable region with gene cassettes	3′ CS*		5′CS*	variable region with gene cassettes
Piglets (*n*=34)	Typical 20 (59)	*int1*	*dfrA1-aadA1* *dfrA12-aadA2* *dfrA7*	*sul1-qacEΔ1*	Typical 7 (20)	*int2*	*dfrA1-sat2-aadA1; estX-sat2-aadA1*
	Atypical 5 (15)	*int1*	*aadA1*	—	Atypical 3 (9)	*int2*	—
		*int1*	—	—			
Sows (*n*=22)	Typical 5 (27)	*int1*	*dfrA1-aadA1* *aadA1* *dfrA7*	*sul1-qacEΔ1*	Typical 9 (41)	*int2*	*dfr1-sat2-aadA1; estX-sat2-aadA1*
	Atypical 14 (64)	*int1*	—	—	Atypical 3 (14)	*int2*	—
Total (*n*=56)	Typical class 1 integrons 25 (45)				Typical class 2 integrons 16 (28)		
	Atypical class 1 integrons 19 (34)				Atypical class 2 integrons 6 (11)		

* 5′CS and 3′CS-conserved segments of integrons.

**Table 3:** Prevalence of elements of transposons Tn7 and Tn21 and the common region ISCR among multidrug-resistant *E. coli* isolated from groups of pigs.

Genetic element	No. (%) of *E. coli* from pigs		

	Piglets (*n*=34)	Sows (*n*=22)	Total (*n*=56)
*tnpA,R merA,R* of Tn21*	12 (35)	10 (45)	22 (39)
*tnpA,R* of Tn21**	5 (15)	2 (9)	7 (12.5)
*merA,R* of Tn21**	12 (35)	4 (18)	16 (29)
*tnsA*,*B,C,D,E* of Tn7	11 (32)	11 (50)	22 (39)
ISCR2	18 (53)	6 (27)	24 (43)

* Complete set of genes of Tn21;

** incomplete set of genes of Tn21.

**Table 4:** Prevalence of Inc plasmid groups among multidrug-resistant *E. coli* isolated from groups of pigs.

Inc group	No. (%) of *E. coli* from pigs		

	Piglets (*n*=34)	Sows (*n*=22)	Total (*n*=56)
K	29 (85)	21 (95)	50 (89)
FIB	31 (91)	14 (64)	45 (80)
I1	28 (82)	10 (45)	38 (69)
FIIA	18 (53)	8 (36)	26 (46)
FIC	8 (23)	7 (32)	15 (27)
Y	5 (15)	8 (36)	13 (23)
P	9 (26)	0	9 (16)
FIA	4 (12)	3 (14)	7 (12)
HI1	2 (6)	0	2 (3)
B/O	1 (3)	0	1 (2)
T	1 (3)	0	1 (2)
