# Supplementary material for: Complexity of Antibiotic Resistance in Commensal Escherichia coli Derived from Pigs from an Intensive-Production Farm
Source: Microbes Environ. 2018 Sep 29;33(3):242–8. doi: 10.1264/jsme2.ME17041 (PMC6167118; doi:10.1264/jsme2.ME17041)
Supplement: Supplementary file 1 [file 33_242_s1.pdf]

Supplement Table S1. Primer characteristics used for Tn7, Tn21 and ISCR elements detection.

Locus	Primer	PCR	Sequence (5'- 3')	Temperature /time of annealing	Product size (bp)	Reference
transposase A Tn7	tnsA-f tnsA-r	multiplex 1	GCGTGGCCAAGGGCATGGTAAA TAGCACGCTGCTCTCCCACTCA	60°C/30 sec	172	This study based on NC_002525.1
transposase B Tn7	tnsB-f tnsB-r		TCCACCTAGACGTGGCGATGCT AGAGACCTTTCGGCGAGGCAGT		367	
transposase C Tn7	tnsC-f tnsC-r		AGCCCAGCGACTGGGATAGCTT CGACCACCTTGCGGTTGCCATA		275	
transposase D Tn7	tnsD-f tnsD-r	multiplex 2	TATGGCAACCGCAAGGTGGTCG ATAGCTTCGTCTCGGCGCTCCT	60°C/30 sec	170	
transposase E Tn7	tnsE-f tnsE-r		TGGTGTCCTAGCAGCGGCAGAT ACCCCATCCGATGCATCCACCT		279	
transposase A Tn21	tnpA-f tnpA-r	multiplex 3	CCAGCACGATGCCTTTGTCGGT GCTTCGCGGTGCAGCTCTGTTA	60°C/30 sec	276	This study based on NC_002525.1GQ293498.1
resolvase R Tn21	tnpR-f tnpR-r		CTTTTGCTCGCCAGCCTCGACA GTATTGCGCTCGCCAAGCAACG		107	
structural gene of mercuty resistance operon	merA-f merA-r	multiplex 4	GCGCCACCACTGATGAGCCAAT GCCACGCCAAGTACGAAGGCAT	60°C/30 sec	279	
regulatory gene	merR-f merR-r		GCCTGGCCGAACACAAGCTCAA GACGCGATCAACGGGCAGGAAA		133	
ISCR1	CR1-F CR1-R	single reaction	TGCTCAACACTGCCAACTTT GTATAGGAAGTATAAACCAC	56°C /30 sec	210	This study based on NC_002525.1GU187357.1
ISCR2	CR2 F- CR2 R-	single reaction	TCTGTTTGACTGACGAGCCT CTTTGGACCGCAGTTGACTC	60°C/ 30 sec	205	This study based on NC_002525.1AF231986.2
ISCR3	CR3 F CR3 R	single reaction	ATCCTCGCCCACTTCGAG GTTGTCAGCAGCCATGCTT	60°C/ 30 sec	203	This study based on NC_002525.1FJ744121.1
